# Cellular and molecular cues of glucose sensing in the rat olfactory bulb

**DOI:** 10.3389/fnins.2014.00333

**Published:** 2014-10-29

**Authors:** Dolly Al Koborssy, Brigitte Palouzier-Paulignan, Rita Salem, Marc Thevenet, Caroline Romestaing, A. Karyn Julliard

**Affiliations:** ^1^Team “Olfaction: From Coding to Memory,” Lyon Neuroscience Center, INSERM U1028-CNRS, University Lyon 1Lyon, France; ^2^Laboratoire d'Ecologie des Hydrosystèmes Naturels et Anthropisés CNRS 5023, University Lyon 1, Bâtiments Darwin C and ForelVilleurbanne, France

**Keywords:** olfactory bulb, glucose sensing, GLUT4, SGLT1, extracellular glucose concentration, feeding state

## Abstract

In the brain, glucose homeostasis of extracellular fluid is crucial to the point that systems specifically dedicated to glucose sensing are found in areas involved in energy regulation and feeding behavior. Olfaction is a major sensory modality regulating food consumption. Nutritional status in turn modulates olfactory detection. Recently it has been proposed that some olfactory bulb (OB) neurons respond to glucose similarly to hypothalamic neurons. However, the precise molecular cues governing glucose sensing in the OB are largely unknown. To decrypt these molecular mechanisms, we first used immunostaining to demonstrate a strong expression of two neuronal markers of glucose-sensitivity, insulin-dependent glucose transporter type 4 (GLUT4), and sodium glucose co-transporter type 1 (SGLT1) in specific OB layers. We showed that expression and mapping of GLUT4 but not SGLT1 were feeding state-dependent. In order to investigate the impact of metabolic status on the delivery of blood-borne glucose to the OB, we measured extracellular fluid glucose concentration using glucose biosensors simultaneously in the OB and cortex of anesthetized rats. We showed that glucose concentration in the OB is higher than in the cortex, that metabolic steady-state glucose concentration is independent of feeding state in the two brain areas, and that acute changes in glycemic conditions affect bulbar glucose concentration alone. These data provide new evidence of a direct relationship between the OB and peripheral metabolism, and emphasize the importance of glucose for the OB network, providing strong arguments toward establishing the OB as a glucose-sensing organ.

## Introduction

Olfaction is a major sensory modality that regulates food consumption and is itself modulated by nutritional status. An increasing body of evidence indicates that this interplay between olfaction and food intake is mediated by direct action of endocrine and metabolic molecules on the olfactory system (Palouzier-Paulignan et al., 2012). Previously, we have demonstrated that feeding states modulate olfactory detection and that hormones related to metabolism and food-intake regulation such as leptin, insulin and ghrelin in turn modulate olfactory sensitivity (Aimé et al., 2007, 2012; Julliard et al., 2007; Tong et al., 2011). These hormones exert their effects by acting directly on mitral cells, the main output neurons of the olfactory bulb (OB) (Fadool et al., 2000; Apelbaum et al., 2005; Hardy et al., 2005). The synaptic connectivity of the OB seems to be closely affected by circulating hormones that reflect energy status, but is also dependent on the uptake and metabolism of glucose (Nawroth et al., 2007; Pain et al., 2011). Studies involving 2-deoxyglucose have shown that glucose consumption increased following odor presentation in olfactory glomeruli, where axons of olfactory sensory neurons (OSN) converge onto dendrites of mitral, periglomerular and tufted cells (Sharp et al., 1975, 1977).

Glucose is the main energy source in the brain. Brain glucose sensors have been identified as specialized neurons that respond to local fluctuations in extracellular glucose levels, and modulate their mean firing rate accordingly (Gonzalez et al., 2008; McCrimmon, 2008). Among all brain regions, glucose-sensing neurons are found primarily in the hypothalamus, the master brain circuit controlling homeostasis, but also in other brain regions such as the brainstem, amygdala, septum, hippocampus and OB (Anand et al., 1964; Oomura et al., 1969; Ritter et al., 1981; Nakano et al., 1986; Shoji, 1992; Balfour et al., 2006; Ren et al., 2009; Tucker et al., 2010, 2013). Central glucose sensors may play a role in (i) communicating information regarding nutrient status to surrounding neurons linked to whole body energy status and (ii) in the maintenance of local energy needs for synaptic function (Routh et al., 2007). In terms of molecular characteristics, central glucose-sensing neurons express several markers such as ATP-dependent potassium channels (K_ATP_), the voltage-dependent potassium channel subfamily member 1.3 (Kv1.3), sodium-dependent glucose transporters (SGLTs), glucose transporters (GLUTs), glucokinase, and AMP kinase (Livingstone et al., 1995; Karschin et al., 1997; Dunn-Meynell et al., 1998; Diez-Sampedro et al., 2001; Kang et al., 2004; O'malley et al., 2006; Tucker et al., 2013).

Numerous measures of brain glucose concentrations and electrophysiological studies have shown that the fluctuation in extracellular fluid glucose concentration is critical to signal the activity of glucose sensing neurons (for review see Routh, 2010). Thus, many studies have focused on determining glucose levels in various regions of the brain following either fasting/feeding or peripheral injections of insulin or glucose to induce normo-, hypo- or hyperglycaemia (Fellows and Boutelle, 1993; Silver and Erecinska, 1994; Hu and Wilson, 1997; Bequet et al., 2000; Poitry-Yamate et al., 2009). In addition to fluctuations in glucose concentrations, changes in expression of glucose receptors and transporters could play a key role in glucose sensing in the peripheral and central nervous systems. The insulin-dependent glucose transporter type 4 (GLUT4) was found in the olfactory system (El Messari et al., 1998). Insulin, known to activate GLUT4 translocation to the plasma membrane (McEwen and Reagan, 2004), is higly present in the OB and its receptor is expressed mainly in glomerular and mitral cell layers (Aimé et al., 2012). Moreover, recent patch-clamp studies have shown that mitral cells change their firing rate in response to changes in glucose concentration, emphasizing the relevance of these cells for processing normal, physiological changes in glucose (Tucker et al., 2010, 2013). Tucker's work allows identifying these neurons as glucose sensors.

It becomes important to understand the cellular and molecular cues underlying OB glucose sensing. In the present paper, with light-dark cycle and feeding state conditions strongly controlled, we used immunofluorescence and Western Blot techniques to map the localization and study the changes in expression of two markers of glucose sensing ability, GLUT4 and SGLT1, in the OB of satiated and fasted rats. We further measured the extracellular fluid glucose concentration ([Gluc]_ECF_) in the OB under two conditions: (i) steady glycaemia state obtained by controlling nutritional states and (ii) dynamic glycaemia state induced by glucose or insulin injections. To ascertain the importance of glucose signaling in the OB neuronal network, [Gluc]_ECF_ was monitored for spatial and temporal fluctuations via continuous recordings in the OBs and cortices of anesthetized rats.

## Materials and methods

### Animals

Experiments were carried out in accordance with the European Community Council Directive of November 24, 1986 (86/609/EEC), for the care and use of laboratory animals. The experimental protocols were approved by the Lyon University Animal Experimentation Committee, and care was taken at all stages to minimize stress and discomfort to the animals. On arrival, adult male Wistar rats (Charles River; 250–350 g) were housed in groups (4–6 rats) in Plexiglas chambers at constant temperature and relative humidity (22 ± 0.5°C and 50 ± 5%, respectively). Animals were acclimated to a 12 h light/12 h dark inverted cycle (lights off at 08:00 a.m.) and had *ad libitum* access to food and water for at least 2 weeks. Three weeks prior to surgical procedures, the rats were gradually habituated to a 22 h/day food restriction schedule in which they had access to food from 08:00 to 10:00 a.m. only. Since daily fluctuations in glycemia and insulin levels are cued by food intake (Kaul and Berdanier, 1975; Sitren and Stevenson, 1978), a single daily meal was imposed to synchronize the circadian variation of glycemia and insulin secretion among the animal cohort. Animals were handled (5 min/d) and weighed daily to assess their adaptation to food restriction.

### Physiological measurements

To characterize the metabolic steady states of the fasted and satiated rats, we measured steady-state concentrations of plasma glucose, plasma insulin, and OB insulin. Peripheral blood glucose level was determined by sampling 5 μL tail blood 1 h before and 2 h after food intake for fasted and satiated rats respectively, and by monitoring glucose levels with a glucose meter (Accu-Chek® Roche, Mannheim, Allemagne/Performa,). Plasma and OB insulin levels were measured in the fasted state at 8:00 a.m. (*n* = 5) or in the satiated state at 12:00 a.m. (*n* = 5). Rats were deeply anesthetized using an intraperitoneal (i.p.) injection of ketamine (Imalgene, 80 mg/kg) and xylazine (Rompun, 10 mg/kg), then rats were euthanized, and their OBs immediately frozen in liquid nitrogen. Trunk blood was collected in heparinized tubes, and the plasma fraction was separated by centrifugation at 2000 g for 5 min. One OB from each rat was conserved for Western blotting and insulin was extracted from the second OB according to the procedure of Baskin and collaborators (Baskin et al., 1983). To determine the influence of the extraction on insulin output, samples with known amounts of insulin were submitted to the same protocol. The mean extraction output was found to be ~40%. Plasma and OB insulin levels were determined using a solid-phase, two-site enzyme immunoassay following the manufacturer's protocol (Mercodia Ultrasensitive Rat Insulin ELISA).

### Immunostaining

Animals were anesthetized (using the same protocol described previously) and euthanized either in the fasted state at 8:00 a.m. (*n* = 4) or the satiated state at 12:00 noon (*n* = 4). Immunofluorescence was performed on fresh frozen brain samples by using a modification of a published method (Julliard and Hartmann, 1998). Brain cryosections were pre-incubated for 15 min with a blocking buffer containing 0.1 M phosphate buffer saline (PBS) (*pH* = 7.4), 3% bovine serum albumin (BSA, Sigma-Aldrich) and 5% normal serum from the host species of the antibodies. The sections were then incubated overnight at 4°C with primary antibodies for the IR (1:50 dilution; Biosource), SGLT-1 (R-16: sc-20584) (1:100; Santa Cruz Biochemicals, Santa Cruz, CA), microtubule-associated protein 2 (MAP2, 1:200, Sigma Aldrich), or Synapsin 1 (1:500; SYnaptic SYstems). Two anti-GLUT4 antibodies were used: a rabbit polyclonal antibody raised against amino acids mapping near the C-terminus of rat GLUT4 (1:100; Millipore), and a mouse monoclonal antibody which recognizes an epitope in the cytoplasmic portion of GLUT4 [1F8] (1:100 abCam). The sections were washed with 0.1 M PBS/3% BSA and incubated for 1 h at room temperature with anti-rabbit IgG, anti-mouse IgG, or anti-goat secondary antisera coupled to Alexa 488 (1:100), Cy3 (1:200; Jackson Immunoresearch), or Cy5 (1/100; Jackson Immunoresearch) respectively. After the final wash with PBS, slides were mounted with Vectashield mounting medium containing DAPI for nuclear staining (Vector Laboratories). Control sections were performed in which the primary antibody was omitted. In addition, to test the specificity of anti-SGLT1 antibody, negative control sections were performed in which the antibody was pre-absorbed with a 25-fold molar excess of the blocking peptide (sc-20584 P, Santa Cruz Biochemicals, Santa Cruz, CA). Images were acquired using a Zeiss Apotome epifluorescence microscope equipped with a digital camera and Axiovision software.

### Western blot analysis

Frozen tissues (OB and cortex) removed from the same rats used for ELISA analysis were homogenized in ice-cold homogenization buffer (Tris 5 mM, EGTA 2 mM, pH 7.4) supplemented with anti-protease cocktail (SIGMA P8340, 10 μl per mg tissue). Protein concentrations were determined by BCA assay according to the manufacturer's recommendation (PIERCE #23225). 100 μ g of proteins were loaded into 7.5% SDS-polyacrylamide gels (SDS-PAGE) and electrophoresed for 1.5 h at 110 V. Proteins were then electro-transferred for 1 h at 300 mA onto a PVDF membrane. Membranes were blocked with 5% milk in TBS containing 0.05% of Tween 20 and incubated overnight with polyclonal antibodies directed against GLUT4 (Millipore 07–1404; dilution 1:250), or SGLT1 (Santa Cruz sc-98974 raised against amino acids 580–657 mapping at the C-terminus of SGLT-1; dilution 1:1000). Equal loading was verified using Ponceau red stain, and by detection of control protein β-actin (Sigma; diultion1:8000). Membranes were washed in 0.05% Tween-PBS buffer and incubated with horseradish peroxidase-conjugated secondary antibody (dilution 1:10000). Signals were detected using the enhanced chemiluminescence detection system (Pierce #32106). Immunoblots were scanned using a desktop scanner (Epson Perfection V350) and Adobe Photoshop. Band intensities were determined using Scion Image (Scion Corporation, USA). SGLT1 positive control was performed on rat kidney extracts. For each glucose transporter, the two different brain areas of the same rat were analyzed in the same Western blot test.

### Glomerular quantification of GLUT4 immunostaining

All GLUT4 measurements were conducted blindly with regards to the metabolic status of rats. All images were acquired within the same exposure time. Quantification of GLUT4 in the glomeruli was performed by measuring the pixel intensity of the GLUT4-Alexa 480 fluorescent signal using the Axiovision densitometric function. All the glomeruli on each image were hand-delimited solely on the basis of DAPI signals. For each animal, three zones were analyzed, each corresponding to a third of the main OB along the rostro-caudal axis (anterior, AZ; intermediate, IZ; and posterior, PZ). In each zone, two frontal sections, separated by 250–300 μm with left and right OBs, were obtained, and each section was divided into 4 sectors corresponding to the dorsomedial (DM), ventromedial (VM), ventrolateral (VL) and dorsolateral (DL) regions of the main OB. An image of each sector was acquired. A total of 384 images were obtained and in total more than 3900 glomeruli were quantified.

The mean densitometric value of GLUT4 labeling was first compared between the zones, then between the sectors. In order to better represent GLUT4 intensity of labeling according to the zones, sectors and feeding states, a colored image plot was constructed using MySql database software and Python script. The glomerular GLUT4 densitometric values were ranked in descending order and were coded with a color scale which illustrates intensities of immunostaining (dark red corresponds to the highest value, blue corresponds to the lowest one). For each sector, a matrix was built with the highest densitometric value placed in the center, and values decreasing in magnitude from the center to the edge of the matrix in a spiral shape, so that the smallest values were located in the most peripheral part of the matrix. The matrices were constructed with a minimum of 130 values, and an average of 160 values. Finally, pseudo-color representations of the matrices were generated, giving a map of the intensity of labeled glomeruli for each sector of the OB.

### Bioprobe measurements of extracellular glucose

#### Surgical procedure

Rats were anesthetized either in the fasted (08:00 a.m.) or satiated (10:00 a.m.) state with urethane (1.5 mg/kg, i.p.). The anesthetized animals were placed in a stereotaxic apparatus and kept on a heating pad. Additional doses of urethane were supplied as needed. The surgical procedure consisted of drilling three burr holes into the skull to expose the lateral region of each OB and in the somatosensory cortex. One glucose-oxidase biosensor was implanted into the lateral part of one OB, within or close to the glomerular layer (coordinates: AP +6.5 mm from bregma, M/L −2.3 mm and D/V −1.0 mm from dura). The second glucose-oxidase biosensor was inserted in the controlateral somatosensory cortex (coordinates: AP −3.5 mm from bregma, M/L +2.3 and D/V −2.3 mm from dura). A control BSA sensor was implanted in the other OB to measure non-specific variations in oxidation current (coordinates similar to glucose biosensors implanted in OB). A reference electrode (Ag/AgCl) was placed into neck muscles during *in vivo* recordings.

#### Preparation of electrochemical sensors

The glucose biosensor uses glucose oxidase and amperometric detection of hydrogen peroxide (Vasylieva et al., 2011). The tip of the probe was coated with glucose oxidase that metabolizes glucose in the extracellular fluid. An oxidation current is thus generated and measured using Neurolabscope software. Each biosensor was connected to a potentiostat, which sends readings of the current generated by glucose in extracellular fluid to a computer. Glucose biosensors have been shown previously to have a range of 0–10 mM with *in vitro* sensitivity of 1.6 ± 0.4 nA/mM (mean ± s.e.m.). To confirm the accuracy of the biosensors, prior to implantation and immediately following testing, we placed each biosensor probe in 0.1 M PBS, connected to the potentiostat, and readings were allowed to stabilize (generally stable within 15–30 min).

#### Selectivity and calibration of biosensors

Prior to surgeries all biosensors were tested for detection of serotonin (5-HT, 20 μ M in PBS; Sigma) and H_2_O_2_ (1 μ M in PBS). Only electrodes exhibiting less than 1.2 μ A.mM^−1^cm^−2^ response for 5-HT were included in the study. *In vitro* calibrations were performed in standard PBS (0.01 M, pH 7.4) and solutions were maintained at a temperature of 36.5°C, comparable to the brain of an anesthetized rat (Zhu et al., 2004). The reference electrode (Ag/AgCl) was placed directly in the solution. After a stable baseline reading, the glucose sensors were calibrated using glucose solutions at different concentrations (0; 0.5; 1; 1.25; and 1.5 mM) to establish the nA/mM ratio. The applied voltage for amperometric studies was +500 mV. Biosensors were calibrated before and after each real-time *in vivo* experiment to ensure the sensitivity remained stable. Quantitative assessments of brain glucose concentrations were obtained by subtracting the non-specific current of the control biosensor (BSA) from the output of the glucose biosensors.

### Real-time *in vivo* measurements

To compare glucose level variations in the OB and cortex to peripheral glucose concentrations, 5 μ l blood samples were collected from the femoral artery at the beginning of the surgery and then every 10 min thereafter. Glucose readings were performed with a glucose meter (Accu-Chek® Roche, Mannheim, Allemagne/Performa). Measurements of extracellular glucose in rat brains started 1 h after the implantation surgery to allow restoration of the blood-brain barrier. Satiated rats (*n* = 9) were anesthetized after food intake and fasted rats (*n* = 8) before food intake. Recordings started once the electrodes were implanted, and lasted for approximately 3 h. Currents obtained after the signal stabilization corresponded to the initial steady state of the animal, i.e., fasted or satiated. To evaluate possible fluctuations of central glucose level during dynamic glycemia conditions, an i.p. injection of glucose (Lavoisier, Paris, France 30%; i.p., 3 g/Kg) to the fasted rats, or a subcutaneous injection of insulin (Sigma, Saint Quentin-Fallavier, France; 7.5 U/mL; subcutaneous, 25 U/Kg) to the satiated rats, were given to promote the effects of acute modifications in peripheral glucose levels on central structures (OB and cortex). An hour later, rats in the induced hyperglycemic state were injected with insulin and rats in the induced hypoglycemic state were injected with glucose. In 1 of 9 satiated rats glucose was monitored in the OB alone, and 3 rats received only insulin injection. In 1 of 8 fasted rats, glucose was monitored only in the OB, and 3 rats received glucose injection only. When recordings ended, rats were euthanized using sodium pentobarbital (i.p. 3 g/Kg).

### Statistical analysis

Data are shown as mean values ± s.e.m. Physiological parameters (glycemia, plasma and OB insulin level) were analyzed using a non-parametric Mann-Whitney test, or Wilcoxon test when data were paired (Statview Software). For Western Blot data, quantification of glomerular GLUT4 immunostaining, and extracellular glucose measurements, statistical analysis were performed by either a Student's *t*-test or one- or Two-Way repeated-measures ANOVA depending on the data set. A Student-Newman-Keuls (SNK) *post-hoc* test was used to complete the analysis when appropriate (Statview software). In all cases, *P* < 0.05 was considered statistically significant.

## Results

### Physiological hallmarks of satiated and fasted rats

Body weight was regularly measured to monitor the influence of restricted access to food on the physiological and nutritional status of rats. Rats started to gain weight only 3 days after the restrictive 2-h access to food had started (data not shown). Glycemia and insulin levels in the blood and the OB were significantly different between satiety and fasting (Table 1, *P* < 0.01, Mann-Whitney test).

**Table 1:** **Effects of feeding states on glycemia, and on insulin levels in the blood and the OB**.

	**Satiated (***n*** = **5**)**	**Fasted (***n*** = **5**)**	***P***
Blood glucose (mM)	10.92 ± 0.46	5.84 ± 0.54	<0.01
Blood insulin (ng/mL)	4.48 ± 0.78	1.55 ± 0.37	<0.01
OB insulin (ng/g)	0.28 ± 0.01	0.13 ± 0.01	<0.01

Means (±s.e.m.) are statistically different between satiated and fasted animals (n = 5/feeding state, P < 0.01, Mann-Whitney test).

### Molecular markers of glucose sensing: GLUT4 and SGLT1

#### Immunohistochemistry in the OB

**Figure 1:**
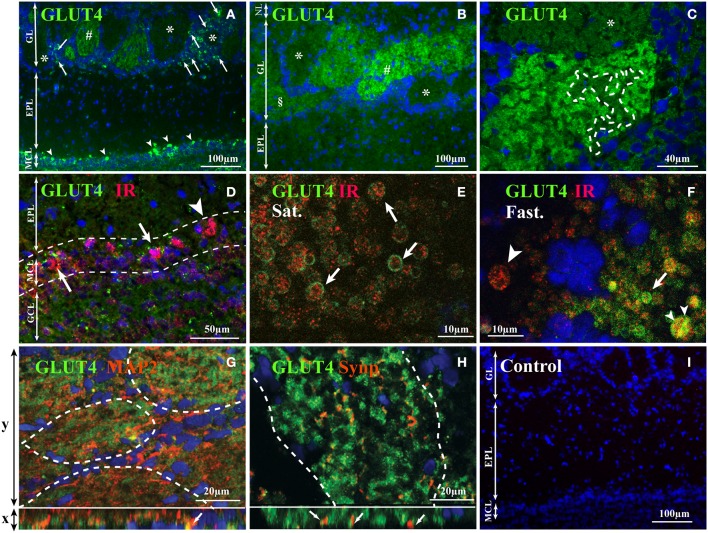
**GLUT4 localization in the OB layers**. Representative GLUT4 immunostaining of the main OB layers of adult rats performed with monoclonal **(A)**, and polyclonal antibodies **(B–H)** merged with the nuclear marker DAPI **(A–C)**, double immunostained with IR **(D–F)**, with MAP2, a dendritic processes marker **(G)** and with Synapsin 1, a presynaptic marker **(H)**. **(A–C)** In the glomerular layer, glomeruli show different staining intensities (strongly labeled: #, slightly labeled: §; not labeled: ^*^). Scattered periglomerular cells (**A**; arrows) around unstained glomeruli (^*^) are GLUT4 positive. Some mitral cells (**A**; arrowhead) are intensively immunostained. **(C)** Within glomeruli the GLUT4 immunostaining is heterogeneous with alternation of highly marked zones and faded zones (delimited by dotted lines). **(D)** In the mitral cell layer, scattered mitral cells co-express GLUT4 and IR (arrows) while others express only IR (arrowhead). **(E,F)** Within glomeruli expressing GLUT4, at high magnification in satiated rats **(E)**, GLUT4 was found enclosing IR clusters located within large processes (**E**; arrows). In fasted rats **(F)**, GLUT4 was either co-localized (**F**: small arrowhead) or not (arrow) in the cytoplasm of these processes. Some processes expressed only IR (large arrowhead). **(G,H)** Immunofluorescence Apotome images of GLUT4, MAP2 and Synapsin 1 within glomeruli. Projection images of consecutive x-y optical sections are shown in the upper large panels. An x-z vertical scanning image of each upper panel is shown in the lower small panel. Green and blue signals indicate GLUT4 and nuclei (DAPI) respectively, and orange indicates MAP2 **(G)** and Synapsin 1 **(H)**. MAP2 can be co-localized with GLUT4 (**G**, arrows in lower small panel; yellow signal) while Synapsin 1 is located close to GLUT4 (**H**, arrows in lower small panel). **(I)** Control section where primary antibodies were omitted: only the nuclear marker DAPI was detected. (NL, nerve layer; EPL, external plexiform layer; GL, glomerular layer; MCL, mitral cell layer; GCL, granular cell layer; MAP2, microtubule-associated protein 2; Syn, Synapsin 1; IR, insulin receptor; Sat., Satiated rat; Fast, Fasted rat).

Using two antibodies, a polyclonal and a monoclonal, a heterogeneous distribution of GLUT4 was observed within the different OB layers. The highest immunostaining was detected in the glomerular layer (GL) and in the mitral cell layer (MCL) (Figures 1A,B). The nerve layer (NL) appeared unstained. GLUT4 immunoreactivity was largely variable within glomeruli and ranged from strongly labeled to non-labeled. Only unlabeled GLUT4 glomeruli were surrounded by distinct GLUT4-labeled periglomerular (PG) cells, while around strongly-labeled glomeruli (Figure 1A) no PG cells expressed GLUT4 (Figure 1C). No specific labeling was detected in control sections in which the primary antibody was omitted (Figure 1I).

GLUT4 is an insulin-sensitive glucose transporter (for review see McEwen and Reagan, 2004); therefore a double-labeling experiment was carried out by combining antibodies directed against GLUT4 and IR (Figures 1D–F). In accordance with our previous data (Aimé et al., 2012), IR was found in glomeruli and mitral cells. Double-labeling revealed that in mitral cells GLUT4 was always localized with IR, but the opposite is not true because some mitral cells expressed only IRs (Figure 1D). In order to study whether the localization of GLUT4 and IR is diet dependent, Z-stack images were performed within the neuropils of strongly-labeled GLUT4 glomeruli in satiated and fasted rats (Figures 1E,F). GLUT4 and IRs were clearly co-expressed in neuronal processes with large diameter (4–5 μm) corresponding to the hallmarks of dendrites of the two types of OB main neurons (mitral and tufted cells) (see for review Shepherd, 2003). In satiated rats, GLUT4 was located on the plasma membrane, while IR was found in the internal part of these neuronal processes, i.e., in the cytoplasm (Figure 1E). In fasted rats, GLUT4 was found mainly in the cytoplasm of these processes, either co-localized with IR or not (Figure 1F). These data imply that glucose fluctuations cause a dynamic distribution of glucose sensing markers.

In order to identify the neuronal compartment (i.e., axonal vs. dendritic) expressing GLUT4 within glomeruli, double-labeling experiments were carried out by combining the anti-GLUT4 antibody with (i) Synapsin 1, a sypnatic vesicle marker (De Camilli et al., 1983) which is located on olfactory terminal axons and on the dendrodendritic synapses within glomeruli (Kasowski et al., 1999) or with (ii) MAP2, a dendritic marker which is highly expressed by the large dendritic trunk of mitral/tufted cells (Bailey et al., 1999; Kasowski et al., 1999; Treloar et al., 1999). GLUT4 appeared co-localized with MAP2 (Figure 1G) but not with Synapsin 1 (Figure 1H), which suggests that it is involved in glucose uptake by the dendrites of mitral cells.

**Figure 2:**
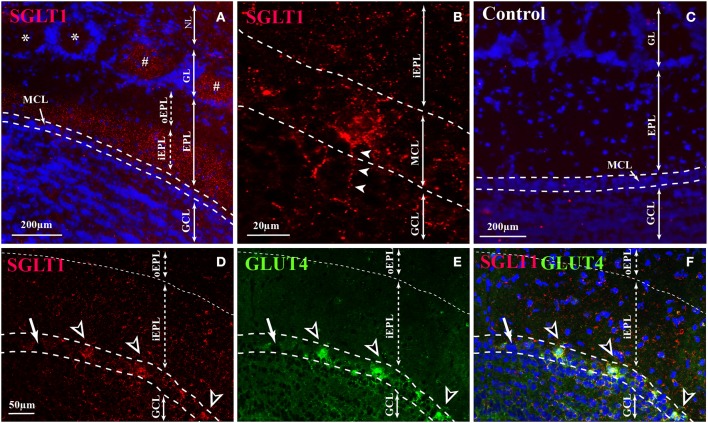
**SGLT1 localization in the OB layers**. Representative SGLT1 immunolocalization in the main OB layers of adult rats **(A,B,D–F)** merged with the nuclear marker DAPI **(A)**, and double immunostained with GLUT4 **(D–F)**. **(A)** In the EPL, SGLT1 is found only in the inner part of the EPL (iEPL) while the outer part (oEPL) does not express SGLT1. In the GL, some (#) glomeruli are stained with SGLT1 but not all (^*^). **(B)** At high magnification, SGLT1 was found in the cytoplasm of some mitral cells and in thin processes located in the GCL which some of them came from mitral cell body (arrowheads). **(D)** Mitral cells which express SGLT1 (black and white arrowheads) are also stained with GLUT4 (**E,F** for merged pictures). Arrow shows mitral cells neither labeled with SGLT1 nor with GLUT4 antibodies. **(C)** The SGLT1 labeling completely disappears in control sections where SGLT1 antibody is absorbed with a blocking peptide. (NL, nerve layer; GL, glomerular layer; EPL, external plexiform layer; iEPL, inner part of external plexiform layer; oEPL, outer part of external plexiform layer; MCL, mitral cell layer; GCL, granular cell layer).

Immunostaining of SGLT1, a transporter of non-metabolized glucose, reveals a layer-specific pattern (Figure 2). The highest staining was observed in the inner part of EPL (iEPL) while the outer part of the EPL (oEPL) was unlabeled (Figure 2A). Some mitral cells and some glomeruli were strongly stained (Figures 2B,D), as neuronal processes located in the glomerular cell layer (GCL) are derived in part from mitral cell bodies (Figure 2B). Double-labeling revealed that SGLT1-positive mitral cells are also labeled with GLUT4 (Figures 2D–F). The specific labeling observed with SGLT1 antibodies had completely disappeared in control sections in which the primary antibody was absorbed with the corresponding blocking peptide (Figure 2C).

#### Effects of feeding states on the location and expression of GLUT4 and SGLT1

**Figure 3:**
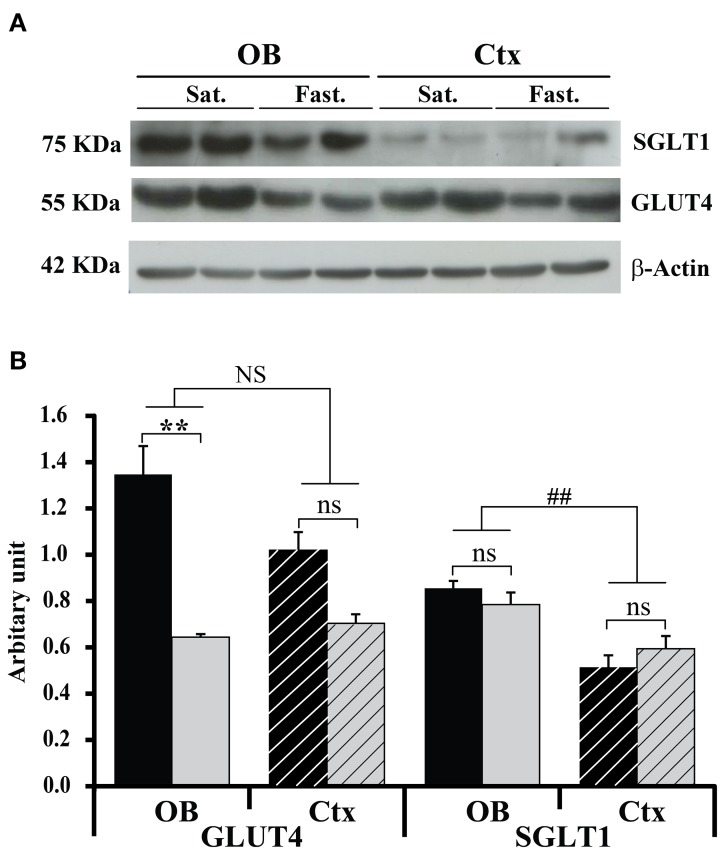
**Feeding states and structure effects on GLUT4 and SGLT1 protein levels**. Representative Western blot **(A)** and quantitative densitometric histograms **(B)** of GLUT4 and SGLT1 protein levels in the OB (full bars) and cortex (hatched bars) of satiated (black bars, *n* = 5) and fasted (gray bars, *n* = 5) rats. Bar graphs represent ratios of the GLUT4 and SGLT1 protein levels after normalization against β –actin. A Two-Way ANOVA reveals significant effect of feeding state for GLUT4 [*F*_(1, 8)_ = 12.8; *P* < 0.01]. A Mann-Whitney test shows that the effect of feeding states satiated (Sat.) vs. fasted (Fast.) on GLUT4 expression is significant only for the OB (^**^P < 0.01). A Two-Way ANOVA reveals significant effect only for the brain area factor [*F*_(1, 8)_ = 19.03, ##*P* < 0.005), and SNK shows that SGLT1 expression in the OB was significantly higher than in the cortex (Ctx). No effect of feeding state is observed for SGLT1 (ns). Error bars represent s.e.m.

The effect of feeding states on GLUT4 and SGLT1 expression within the OB and the somatosensory cortex was analyzed by Western blot (Figure 3). Total tissue GLUT4 and SGLT1 were analyzed using the total protein recovery from the OBs and somatosensory cortices of 5 satiated and 5 fasted rats. A Two-Way ANOVA on GLUT4 expression, with feeding state and brain area as factors, revealed a significant effect of feeding state [*F*_(1, 8)_ = 12.8, *P* < 0.01] and no effect of brain area [*F*_(1, 8)_ = 2.55, *P* = 0.15] despite a significant interaction between these two factors [*F*_(1, 8)_ = 5.3, *P* < 0.05]. GLUT4 was significantly higher in satiated rats than in fasted rats (*P* < 0.01, Mann-Withney test), while in the somatosensory cortex, GLUT4 was not significantly different in the two animal groups (*P* = 0.12, Figure 3B). Thus, chronic restricted feeding up-regulates GLUT4 expression in the OB but not in the somatosensory cortex.

SGLT1 positive control performed by using rat kidney tissue extract revealed that SGLT1 antibodies bound to a polypeptide band at about 75 KDa (data not shown). Conversely to GLUT4, there was no interaction between brain areas and feeding states for SGLT1. SGLT1 expression was however, significantly different between the two brain areas [*F*_(1, 8)_ = 19.03, *P* < 0.005], but independent from the feeding state. A *post-hoc* test (SNK) showed further that SGLT1 expression was higher in the OB (Figure 3B).

**Figure 4:**
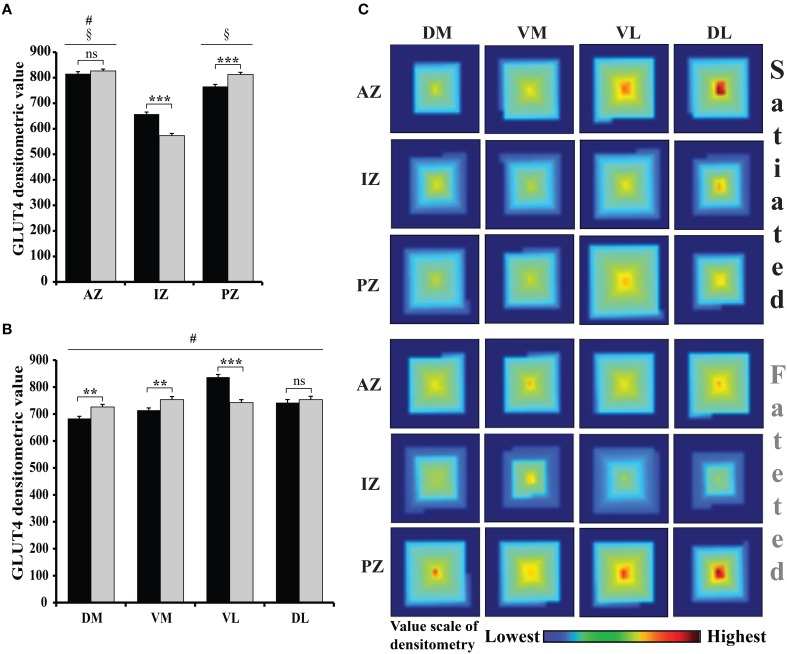
**GLUT4 distribution and the effect of feeding state on GLUT4 in the glomerular layer**. **(A,B)** The bar graphs represent an arbitrary unit of the GLUT4-Alexa 488 densitometric value (mean ± s.e.m.) quantified within each glomerulus of frozen OB sections of satiated (black bars) and fasted (gray bars) rats. Graph **(A)** compares the different zones, while **(B)** compares the different regions. Repeated measures ANOVA revealed significant effects of zones (ANOVA, *P* < 0.0001) and regions (ANOVA, *P* < 0.001) with high interaction with feeding states (*n* = 8 including 4 fasted rats and 4 satiated rats). **(A)** Zone Effect: The mean GLUT4 densitometry was significantly higher in the anterior zone (AZ) compared to the posterior zone (PZ) which was higher than the intermediate zone (IZ) (SNK, compared to PZ #*P* < 0.05, compared to IZ §*P* < 0.05). Effect of feeding state (satiated vs. fasted) on GLUT4 densitometry was significant in the IZ and PZ but not in the AZ (*t*-test, ^***^P < 0.0001; ^**^P < 0.01; ns: non-significant). **(B)** Region Effect: GLUT4 densitometric values in the dorsomedial (DM), ventromedial (VM), dorsolateral (DL) and ventrolateral (VL) regions were significantly different from each other (SNK, #). A significant effect of feeding state was observed in all regions except for VL (Student's *t*-test, ^**^P < 0.01 for DM and VM regions; ^***^P < 0.001 for VL region). **(C)** Feeding state effect on GLUT4 expression in glomeruli. In this map, each square corresponds to one region and one zone in the fasted or satiated state. The square is built from a spiral pattern matrix in which the highest glomerular densitometric value is placed in the center of the matrix and values decrease in magnitude from the center to the edge of the matrix. In satiated rats the glomeruli exhibiting the highest GLUT4 staining are observed in dorsolateral (DL) region of the AZ, while in fasted rats these glomeruli are observed in the dorsolateral (DL) region of the PZ.

Regionalization of GLUT4 in the glomeruli of adult fasted and satiated rats was studied by two different immunohistochemistry procedures: (i) using a rabbit anti-GLUT4 primary antibody, and (ii) omitting the primary antibody. A Three-Way repeated-measure ANOVA with the feeding states, zones (AZ, IZ, and PZ), and regions (DM, VM, VL, and DL) as factors of densitometric values revealed a significant effect of feeding states [*F*_(1, 236)_ = 3.8, *P* < 0.05], zones [*F*_(2, 472)_ = 205.95, *P* < 0.0001], and regions [*F*_(3, 708)_ = 63.46, *P* < 0.0001]. A significant interaction was also noted between feeding states and zones [*F*_(2, 472)_ = 40.88, *P* < 0.0001] and between feeding states and regions [*F*_(3, 708)_ = 33.47, *P* < 0.0001]. Analyzing the OB zones (Figure 4A), AZ had a significantly higher mean densitometric value than PZ, which had a significantly higher mean densitometric value than IZ (SNK *post-hoc* tests). Fluorescence density in every OB region (Figure 4B), was significantly different than every other, the VL region having the highest mean densitometric value. Since interactions were found, the feeding state effect was compared for each zone and each region using a *t-*test (Figure 4A). Feeding state had an effect on GLUT4 density in IZ and PZ (*t* = 7.044, *P* < 0.0001; *t* = 3.91, *P* < 0.0001, respectively) and no effect in AZ (*t* = 0.97, *P* = 0.33). Feeding state had also an effect on GLUT4 density in three regions (DM, VM, and VL) (*t* = 3.36, *P* < 0.001; *t* = 2.76, *P* < 0.01; *t* = 6.50, *P* < 0.0001 respectively, Figure 4B) and no effect in DL region (*t* = 0.66, *P* = 0.50). To summarize, the most stained glomeruli were located mainly ventrally and dorsally of the lateral AZ in satiated rats, and of the PZ in fasted rats as shown on the spiral matrix in Figure 4C. We conclude that a shift in GLUT4 density occurred along the anteroposterior axis in the VL and DL regions.

### Real time *in vivo* monitoring of extracellular fluid glucose in the OB and the cortex

#### During steady states

**Figure 5:**
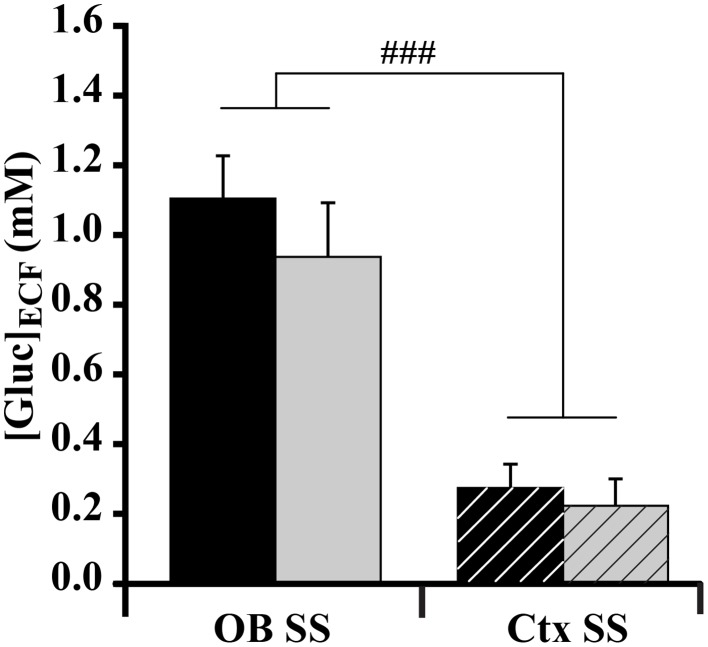
**Effect of feeding state on glucose level ([Gluc]_ECF_) measured at the steady state (SS) in the OB and cortex (Ctx)**. Bar graphs represent the mean (±s.e.m.) of [Gluc]_ECF_ measured in the OB (solid bars) and Ctx (hatched bars) in satiated (black bars) and fasted (gray bars) rats. A significant difference is observed between OB and Ctx [ANOVA, *F*_(1.32)_ = 41.837, ###*P* < 0.0001]. SNK *post-hoc* test reveals that the OB has a significantly higher glucose level than the Ctx. No effect of feeding state (satiated vs. fasted) was obtained (ANOVA, *P* > 0.05) (satiated *n* = 9; fasted *n* = 8).

For both satiated and fasted rats, each ([Gluc]_ECF_) was measured in the OB glomerular layer and in the somatosensory cortex simultaneously. Two weeks prior to the experimental study, animals were habituated to a regular 2-h feeding/22-h starvation schedule in which feeding started at a specific daily time. At the beginning of the experiment, the metabolic status of rats was considered to be the steady state, as satiety and hunger were maintained for several hours. Glycemia was monitored to verify the metabolic state of rats. Blood glucose was significantly different between fasted and satiated animals (satiated rats = 10.633 ± 0.38 mM; fasted rats = 6.287 ± 0.64 mM; *t* = 5.9, *P* < 0.0001, non-repeated-measures *t*-test). A Two-Way ANOVA was performed with brain areas (OB and cortex) and feeding states as factors. For the factor “brain areas” a significant effect on [Gluc]_ECF_ was observed during the initial steady state [*F*_(1.32)_ = 41.837, *P* < 0.0001] as [Gluc]_ECF_ was higher in the OB than in the cortex (Figure 5, *OB* = 1.026 ± 0.1 mM; Ctx = 0.243 ± 0.053 mM, SNK *post-hoc* tests). No effect was observed for the factor “feeding states,” even though glycemia was significantly different between rats in satiated and fasted steady states (Table 1).

#### During dynamic states following insulin and glucose injections

**Figure 6:**
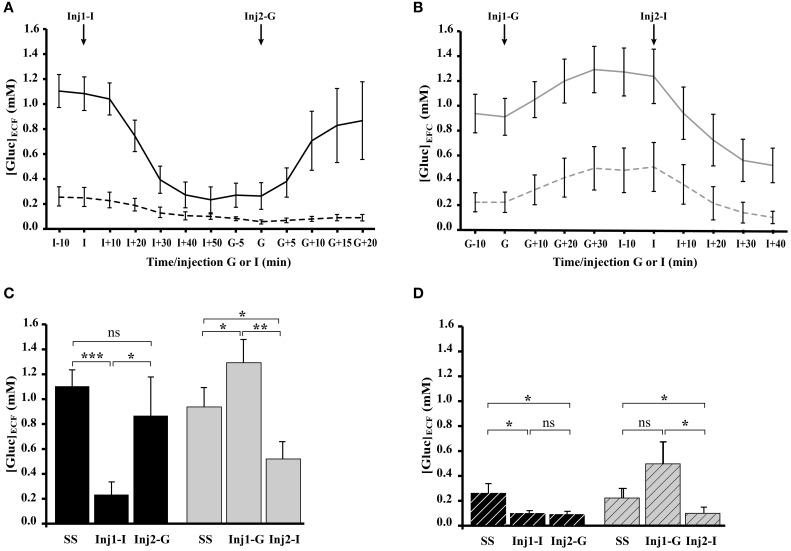
**Effect of glucose and insulin injections on [Gluc]_ECF_ recorded simultaneously in the OB and cortex (Ctx) of satiated and fasted rats**. **(A,B)** Composite figure compiled from *in vivo* real time recordings of [Gluc]_ECF_, performed simultaneously in OB (filled lines) and Ctx (dotted lines) of satiated (**A**, *n* = 9) and fasted (**B**, *n* = 8) rats. The two groups of rats received two injections (Inj1 and Inj2) of insulin and glucose in an opposite order: for satiated rats Inj1 = insulin (Inj1-I) and Inj2 = glucose (Inj2-G), for fasted rats Inj1 = glucose (Inj1-G) and Inj2 = insulin (Inj2-I). **(C,D)** Bar graphs represent the mean (±s.e.m.) of [Gluc]_ECF_ measured in the OB (**C**, filled bars) and Ctx (**D**, hatched bars) of satiated (black bars) and fasted (gray bars) rats. Repeated measures ANOVA performed in satiated and fasted rats reveals a significant effect of brain areas (OB and Ctx; *P* <0.01, *P* < 0.05, respectively) and conditions (Steady State: SS; Inj1; Inj2; *P* < 0.0001, *P* < 0.05, respectively for OB and Ctx). Student's paired *t*-tests performed on [Gluc]_ECF_ measured in the OB during SS and after Inj1 or Inj2 **(C)**, reveal that in satiated rats, only Inj1-I modifies significantly the OB [Gluc]_ECF_ compared to the SS (^***^P < 0.0001). In fasted rats the three measures (SS, Inj1-G and Inj2-I) are significantly different from each other (SS vs. Inj1-G and SS vs. Inj2-I; ^*^P < 0.05; Inj1-G vs. Inj2-I, ^**^P < 0.005). In the Ctx **(D)** the Student's paired *t*-test showed no significant difference between [Gluc]_ECF_ measured in both groups (satiated or fasted) except between the SS and Inj2-I in fasted rats (^*^P < 0.05). Error bars represent s.e.m.

Real time *in vivo* recordings of [Gluc]_ECF_ were performed simultaneously in the OB glomerular layer and in the somatosensory cortex of satiated (Figure 6A) and fasted (Figure 6B) rats. Satiated rats received insulin injection first (Inj1-I) then glucose (Inj2-G) and the order was reversed for fasted rats (Inj1-G, Inj2-I). Insulin injections induced a substantial decrease in extracellular glucose levels in the OB but not in the cortex. Glucose injections induced a less-pronounced increase in glucose concentration. A Two-Way repeated-measures ANOVA with brain areas (OB and Cortex) and conditions (steady-state, Inj1, and Inj2) as factors was performed for each group (satiated and fasted). Results showed a significant effect of brain areas [*F*_(1, 9)_ = 7.53, *P* < 0.05; *F*_(1, 10)_ = 10.90, *P* < 0.01, respectively] and of conditions [*F*_(2, 18)_ = 4.25, *P* < 0.05; *F*_(2, 20)_ = 14.74, *P* < 0.0001, respectively]. In the OB of satiated rats (Figure 6C, black bars), [Gluc]_ECF_ decreased significantly after Inj1-I from 1.10 ± 0.13 mM in steady state to 0.23 ± 0.1 mM (*P* < 0.0001, Student's paired *t*-tests) and increased after Inj2-G to 0.87 ± 0.31 mM (*P* < 0.05, Student's paired *t*-tests). Noteworthy that glucose injection was able to restore a steady satiated state because the last [Gluc]_ECF_ level reached was not significantly different compared to the initial one (0.87 ± 0.31 mM vs. 1.10 ± 0.13 mM, *P* = 0.16). In the OB of fasted rats (Figure 6C, gray bars), [Gluc]_ECF_ in the steady state (0.93 ± 0.15 mM, Student's paired *t*-test), and following Inj1-G (1.29 ± 0.18 mM) and Inj2-I (0.51 ± 0.14 mM) were significantly different each time (Figure 6C, *P* < 0.005, Student's paired *t*-test).

In the cortex (Figure 6D) insulin injections significantly modified extracellular glucose concentration in satiated (steady state: 0.26 ± 0.08 mM, Inj1-I: 0.1 ± 0.02, *P* < 0.05, Student's paired *t*-test) and fasted rats (steady state: 0.22 ± 0.08 mM, Inj1-G: 0.5 ± 0.17 mM, and Inj2-I: 0.1 ± 0.05 mM, *P* < 0.05, Student's paired *t*-test). Glucose injection had no significant effect (Inj2-G vs. Inj1-I and Inj1-G vs. Inj2-I in satiated and fasted rats respectively).

## Discussion

The idea that the brain senses glucose emerged several decades ago, but until recently this property was thought to be confined mainly to the hypothalamus. New data show that mitral cells in the OB are glucose sensors because they modulate their firing frequency in response to changes in glucose concentration (Tucker et al., 2010, 2013). However, it is necessary to go further in order to identify the molecular cues involved in OB glucose sensing. In this study, two markers of glucose sensing (GLUT4 and SGLT1) were found in the OB. Moreover, the effects of feeding states on the expression of these two proteins were demonstrated and *in vivo* glucose measurements were recorded simultaneously in two brain areas to support the idea that metabolic status can modify glucose levels in the OB and not in the somatosensory cortex.

### GLUT4 and SGLT1 are differently mapped in OB layers

GLUT4 was heterogeneously distributed within the glomerular neuropil, and showed a large range of staining from strongly-labeled to unlabeled glomeruli. Within highly labeled glomeruli, GLUT4 was mainly present in large neuronal processes and was co-localized with MAP2, thus disclosing that GLUT4 was mainly present in primary dendrite endings of mitral/tufted cells or in PG cells. This result confirms previously-reported dendritic and post-synaptic GLUT4 locations (Leloup et al., 1996), and reinforces the assumption that GLUT4 which is highly present at the synaptic level plays a role in neurotransmission (Alquier et al., 2006). Conversely, around unlabeled glomeruli, GLUT4 was only present on PG cells. In the glomerular layer, GLUT4-dependent glucose sensitivities of mitral cells and inhibitory PG cells may participate in modulating synaptic transmission of odor-related stimuli. It will be interesting in future experiments to study whether the spatial organization of GLUT4 follows odor mapping in relationship to the molecular features of odorants (Uchida et al., 2000; Leon and Johnson, 2003; Mori et al., 2006).

SGLT1, known to transport non-metabolized glucose, was mainly observed in the iEPL, i.e., in the region where lateral dendrites of one subpopulation of mitral cells interact with deep granular inhibitory interneurons through dendrodendritic reciprocal synapses (Mouradian and Scott, 1988). The immunostaining observed in mitral cells suggests that in the iEPL, SGLT1 is located on the lateral dendrites of mitral cells. In the OB, SGLT1 immunostaining appears to be strata-specific as previously described for other molecules in the EPL (Veh et al., 1995; Ozaita et al., 2002; Imamura et al., 2006; Choi et al., 2010; Lepousez et al., 2010; Stanic et al., 2010). The localization of SGLT1 at the level of the mitral-granule microcircuits which are known to be implicated in odor discrimination (Yokoi et al., 1995; Lledo et al., 2005; Abraham et al., 2010) provides an anatomical basis for a specific neuromodulatory role of SGLT1 at this level of olfactory processing.

### GLUT4 and IR subcellular distributions are modulated by the feeding state

A strong degree of overlap was present in the OB between GLUT4 and IR, as expected for an insulin-dependent glucose transporter. However, our data show that their respective subcellular distribution depends on the feeding state of the animal. Within strongly labeled glomeruli, GLUT4 was mainly expressed on the plasma membrane of neuronal processes in satiated rats, whereas it was observed in the cytoplasm of fasted ones. IR showed a reverse distribution, i.e., cytoplasmic in satiated rats and mostly on the plasma membrane in fasted rats. This compartmentalization is in agreement with a GLUT4 translocation to the membrane, mediated by an increase in insulin (here physiologically induced by satiation) as described previously in skeletal muscles and adipocytes (for review see Watson and Pessin, 2001) and more recently in the hippocampus (Grillo et al., 2009). It will be interesting in future experiments to study this trafficking in details.

### Expression and mapping of GLUT4 but not SGLT1 are modulated by the feeding state in the OB

Insulin level and GLUT4 expression were modulated by the feeding state in the OB but not in the cortex (insulin level and GLUT4 expression were increased in satiated rats compared to fasted rats). These results are consistent with the ideas (i) that insulin level is one of the factors regulating GLUT4 translocation (Sivitz et al., 1990; Vannucci et al., 1998) and (ii) that GLUT4 expression is region specific (Alquier et al., 2001, 2006). GLUT4 expression is also dependent on the feeding state since glomerular GLUT4 mapping shifted from AZ in satiated rats to PZ in fasted rats. We suggest that glomerular mapping of GLUT4 in relationship to food intake could dynamically and temporally change odor representation as olfactory learning do through centrifugal innervations (Martin et al., 2004; Salcedo et al., 2005; Mouret et al., 2009). Olfactory processing and mapping is thus expected to be regulated in the OB depending on the behavioral context and physiological state of the animal.

Unlike GLUT4, the expression of SGLT1 was not affected by the feeding state in the OB. The modulation of this glucose sensor expression in the OB seems to be restricted to pathological contexts such as obesity. Indeed, we have recently demonstrated an up-regulation of SGLT1 in the OB of insulin-resistant and obese Zucker *fa/fa* rats (Aimé et al., 2014). In various pathological contexts like obesity, epilepsy or ischemia (Poppe et al., 1997; Elfeber et al., 2004; Aimé et al., 2014) up-regulation of SGLT1 in specific brain areas is essential to compensate impairment in GLUTs function and to preserve glucose-sensing function (Poppe et al., 1997; Elfeber et al., 2004; Yu et al., 2010, 2013; Aimé et al., 2014).

### At steady and dynamic glycemia states, [Gluc]_ECF_ is differently affected in the OB and the somatosensory cortex

Just after surgically implanting glucose electrodes, glycemia was measured in order to characterize the metabolic status of the animal. At steady state, glycemia was higher in satiated (10 mM) than in fasted (6.2 mM) rats as expected, and the levels obtained are in accordance with those measured by Silver and Erecinska (1994).

At steady glycemia states, in non-stimulated anesthetized rats, glucose concentration measured in the OB using highly sensitive biosensors matched previously reported values (from 0.7 to 2.5 mM) in brain areas of euglycemic rats (for review see Routh, 2010). In the somatosensory cortex, [Gluc]_ECF_ appeared lower (0.243 mM) and in accordance with the range (0.7 mM) previously reported (Vasylieva et al., 2011). This data confirms that [Gluc]_ECF_ is structure specific and compartmentalized according to the brain area studied and the level of neural activity (McNay and Gold, 1999; McNay et al., 2001). The higher [Gluc]_ECF_ in the OB is consistent with its vascular properties: a high capillary network density (Chaigneau et al., 2007) combined with a highly permeable blood brain barrier (BBB) (Ueno et al., 1996). At steady glycemia states, [Gluc]_ECF_ in the OB and in the somatosensory cortex was independent of the feeding states. This could be due to adjustments of the glucose transport capacity at the BBB in response to brain metabolic rate and glucose availability (for review see Bradbury, 1993; Leybaert, 2005; Banks, 2006). For example, BBB permeability has been shown to be 2-fold higher in starved rats than in fed animals (Hargreaves et al., 1986). The main transporter of glucose from the vessels toward brain cells (Pardridge et al., 1990) is the glucose transporter type 1 (GLUT1) which has been identified in OB and cortex vessels (Dobrogowska and Vorbrodt, 1999). By adapting its expression, GLUT1 could compensate for a decrease or an increase in glucose levels during hypoglycemia (Simpson et al., 1999) and hyperglycemia respectively.

During dynamic glycemia fluctuations, [Gluc]_ECF_ is much more impacted in the OB than in the cortex. In the OB, whatever the order of injection (insulin then glucose or the reverse), [Gluc]_ECF_ significantly changed within a physiological range of 0.3–1.3 mM as it was observed in the ventromedial hypothalamic nucleus (De Vries et al., 2003) a well known glucose sensing area (for review see Burdakov et al., 2005). In the somatosensory cortex, insulin injections induced minor [Gluc]_ECF_ modifications (lower than in the OB) and glucose injections had no effect. The difference in [Gluc]_ECF_ variations between OB and cortex could be due to differences in BBB permeability (Ueno et al., 1996) and/or in blood flow within the two brain areas, i.e., higher in OB than in cortex (Yang et al., 1996). The higher sensitivity of the OB to peripheral glucose fluctuations could serve to adapt its function to the feeding behavior.

## Conclusion

**Figure 7:**
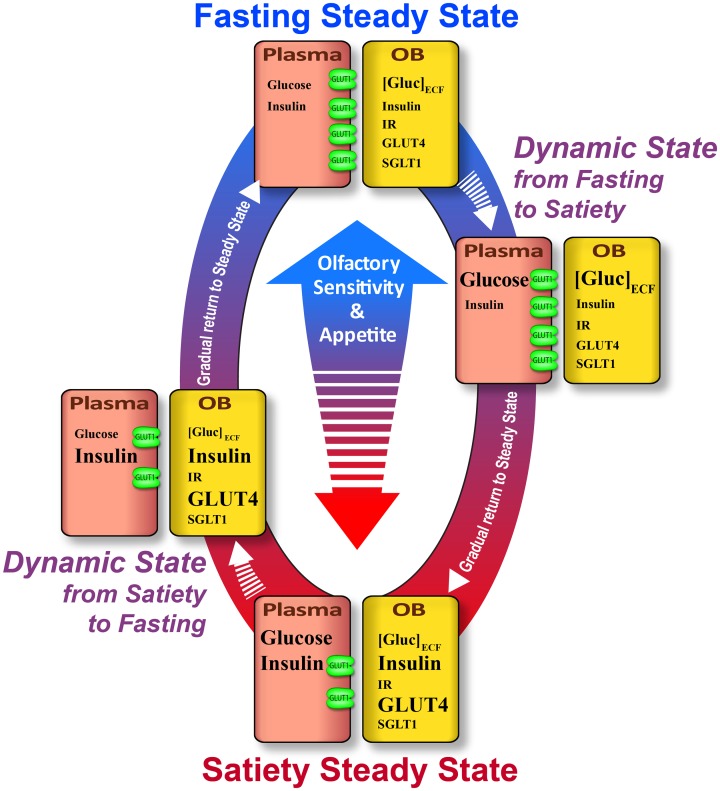
**Schematic summary of variations of the glucose sensing cues in the plasma and OB according to the feeding state**. Satiety steady state: In the plasma, glucose and insulin levels are high. In the OB, [Gluc]_ECF_ is lower while insulin level is high. The lower glucose concentration in the OB could be explained by low GLUT1 expression on endothelial cells. GLUT1 allows delivery of a small quantity of glucose into the OB compared to the high level of plasmatic glucose. In the OB, although IR expression is unchanged, the high level of insulin induces the increase of GLUT4 expression and translocation to the plasma membrane which enhances glucose sensitivity of neurons that express GLUT4. Together these molecular alterations concomitantly decrease appetite and olfactory sensitivity. Dynamic state from satiety to fasting: In the plasma, glucose level decreases due to the increase in insulin level. In the OB, [Gluc]_ECF_ diminishes following glycemic decrease. GLUT1 expression in the endothelial cells of BBB is not predicted to change by this fast plasmatic glucose decrease (30 min–max). In this dynamic state, GLUT4 will likely still be highly expressed on the plasma membrane neurons, thus the glucose sensitivity of neurons will still be important. Since SGLT1 expression is not modulated by the feeding state, this molecular marker of glucose sensing could be involved in dynamic state when OB [Gluc]_ECF_ is altered. Fasting steady state. Glucose and insulin levels in the plasma are low. In the OB, [Gluc]_ECF_ is stable while insulin is reduced. This steady [Gluc]_ECF_ could be due to an adaptive mechanism which takes place in the BBB to preserve brain homeostasis of [Gluc]_ECF_ i.e., GLUT1 overexpression which maintains glucose inflow from plasma to the brain despite the low glycemia. GLUT4 expression is decreased following a decrease in OB insulin level. Moreover, in that state this insulin-dependent glucose transporter relocates to the cytoplasm of neurons. Although OB [Gluc]_ECF_ is similar in the two steady states, the GLUT4-induced sensitivity is reduced during fasting steady. Together these molecular changes could lead to increasing appetite and olfactory sensitivity. Dynamic state from fasting to satiety: In the plasma, glucose concentration is increased while insulin level is still low. [Gluc]_ECF_ in the OB is high while insulin is reduced. The increase in OB [Gluc]_ECF_ is enabled by the high expression of GLUT1 by endothelial cells which increases the influx of glucose from the plasma to the OB. In this dynamic state, the increase in plasma glucose is too fast to induce the down regulation of GLUT1 which will take effect in the following steady state. As pointed for the previous dynamic state (from satiety to fasting), SGLT1 expression is not modulated by the feeding state, so its role could be involved in that dynamic state when OB [Gluc]_ECF_ is modified. Neither IR protein expression and mRNA (Aimé et al., 2012) nor SGLT1 expression change in relationship to the feeding state. Nevertheless, these receptors and glucose transporters could play an important role in OB glucose sensing especially when the concentration of their ligands (insulin and glucose respectively) change during the different states (steady or dynamic states). Arrows: Their length indicates the delay between two successive states. Molecules names: The font is related to their concentration.

In the present study we show that [Gluc]_ECF_, insulin level, expression, translocation and distribution of GLUT4 in the OB are influenced by steady or dynamic feeding states while SGLT1 expression is not regulated in a metabolically-dependent manner. Given that central glucose sensing neurons assume different roles (see Routh et al., 2007), we suggest that GLUT4 and SGLT1 in the OB are involved in different glucose sensing functions. Insulin dependent GLUT4 could interfere in detecting and integrating changes in whole body energy balance, while SGLT1 would be implicated in detecting and regulating glucose availability which is necessary to meet the demands of local synaptic activity. Thus, GLUT4, in addition to peptides involved in food intake regulation, could be responsible for the difference in olfactory sensitivity observed between satiated and fasted rats (Aimé et al., 2007). This hypothesis schematized in Figure 7, is consistent with a recent study showing that impairment of GLUT function could alter olfactory sensitivity in obese *fa/fa* rats compared to their lean counterparts (Aimé et al., 2014). Concerning SGLT1, we suggest that this glucose sensing marker could be involved in maintaining local energy reserves to support bulbar synaptic function known to require very high budget of energy (Nawroth et al., 2007).

## Authors contributions

All experiments were performed in the Centre de Recherche en Neurosciences de Lyon (CRNL). Inserm U1028-CNRS 5292- UCBL1, Team - Olfaction: From Coding to Memory, Lyon, France. Dolly Al Koborssy and A. Karyn Julliard were responsible for the conception and design of the experiments; Dolly Al Koborssy, Caroline Romestaing, Rita Salem, and Brigitte Palouzier-Paulignan, were responsible for the collection of the data; A. Karyn Julliard and Dolly Al Koborssy were responsible for data analysis and interpretation; and Marc Thevenet. designed the software used in analysis., Dolly Al Koborssy, Brigittte Palouzier-Paulignan and A. Karyn Julliard drafted the article and revised it critically for important intellectual content. All authors approved the final version of the manuscript.

### Conflict of interest statement

The authors declare that the research was conducted in the absence of any commercial or financial relationships that could be construed as a potential conflict of interest.
